# Follow-up in patients with a burn-related emergency department visit: a feasibility study

**DOI:** 10.1186/s41038-017-0100-1

**Published:** 2017-11-08

**Authors:** H. Goei, B. F. M. Wijnen, S. Mans, M. A. C. de Jongh, C. H. van der Vlies, S. Polinder, N. E. E. van Loey, M. E. van Baar

**Affiliations:** 1 Association of Dutch Burn Centres, Burn Centre, Maasstad Hospital, PO Box 9100, 3007 AC Rotterdam, the Netherlands; 2 Department of Plastic, Reconstructive and Hand Surgery, MOVE Research Institute, VU University Medical Centre, Amsterdam, the Netherlands; 3 Department of Health Services Research, CAPHRI School of Public Health and Primary Care, Maastricht University, 6200 MD Maastricht, the Netherlands; 4 Trauma Centre Brabant, Elisabeth-TweeSteden Hospital, Tilburg, the Netherlands; 5 Burn Centre, Maasstad Hospital, Rotterdam, the Netherlands; 6 Department of Public Health, Erasmus Medical Centre, Rotterdam, the Netherlands; 7 Association of Dutch Burn Centres, Red Cross Hospital, Beverwijk, the Netherlands

**Keywords:** Burns, Emergency department, Recruitment strategy, Response rate, Health care survey methods

## Abstract

**Background:**

Data on epidemiology, costs, and outcomes of burn-related injuries presenting at emergency departments (EDs) are scarce. To obtain such information, a questionnaire study with an adequate response rate is imperative. There is evidence that optimized strategies can increase patient participation. However, it is unclear whether this applies to burn patients in an ED setting. The objective of this feasibility study was to optimize and evaluate patient recruitment strategy and follow-up methods in patients with burn injuries presenting at EDs.

**Methods:**

In a prospective cohort study with a 6-month follow-up, patients with burn-related injuries attending two large EDs during a 3-month study period were included. Eligible patients were quasi-randomly allocated to a standard or optimized recruitment strategy by week of the ED visit. The standard recruitment strategy consisted of an invitation letter to participate, an informed consent form, a questionnaire, and a franked return envelope. The optimized recruitment strategy was complemented by a stamped returned envelope, monetary incentive, sending a second copy of the questionnaire, and a reminder by telephone in non-responders. Response rates were calculated, and questionnaires were used to assess treatment, costs, and health-related quality of life.

**Results:**

A total of 87 patients were included of which 85 were eligible for the follow-up study. There was a higher response rate at 2 months in the optimized versus the standard recruitment strategy (43.6% vs. 20.0%; OR = 3.1 (95% CI 1.1–8.8)), although overall response is low. Non-response analyses showed no significant differences in patient, burn injury or treatment characteristics between responders versus non-responders.

**Conclusions:**

This study demonstrated that response rates can be increased with an optimized, but more labor-intensive recruitment strategy, although further optimization of recruitment and follow-up is needed. It is feasible to assess epidemiology, treatment, and costs after burn-related ED contacts.

**Electronic supplementary material:**

The online version of this article (10.1186/s41038-017-0100-1) contains supplementary material, which is available to authorized users.

## Background

Worldwide, several epidemiological studies on emergency department (ED) treatments of burn injuries are available [1–4]. These studies focus on incidence rates [1, 3, 4] and trends [1, 4] of burn-related ED visits. In contrast, data on medical or societal costs after burns treated at an ED are scarce. A recent review on costs and cost-effectiveness of burn care revealed a substantial number of studies (*n* = 156) and predominantly costs studies (*n* = 153) [5]. However, data on costs including EDs were limited and showed a broad range (between 88 USD for minor burns and 751 USD for the most severe burn category) [6–8]. Although individual costs may be limited, societal costs can be substantial due to high volumes of burn injuries presented at ED and loss in economic productivity. In addition, to our knowledge, no studies exist into health-related quality of life (HRQOL) after burns treated at an ED. Other previously conducted studies on HRQOL after specialized burn care and after general injuries presented at an ED proved to be feasible [9–12]. However, response rates were low (37%–43%) [9, 10].

To obtain reliable information, the response rate is crucial for the efficiency of the study. A low response rate requires more patients to be included, and selective non-response can bias outcomes. Several systematic literature reviews are available which examined effective strategies to increase response rate both specific to the healthcare setting and postal questionnaires in general [13–15]. The use of colored ink, information brochure, stamped return envelopes, and a monetary incentive have all been associated with a higher response rate. Furthermore, sending non-responders a second copy of the questionnaire was shown to increase response rates [13–15].

The ED is considered the most suitable place for recruitment of incident injury patients in general. However, difficulties in recruitment may arise related to this clinical setting (i.e., large number of staff, small time window present) and the type of patients (low-frequency injuries). In addition, privacy legislation and ethical and research governance influence possibilities for recruitment [16].

In our study, emphasis was put on the optimization and evaluation of patient recruitment strategies and follow-up methods by comparing the effects of an optimized recruitment strategy to a standard strategy. The objective of this feasibility study was to improve and evaluate patient recruitment strategy and follow-up methods.

## Methods

### Study design, setting, and population

A prospective cohort study with a 6-month follow-up was conducted. All patients with burn-related injuries attending the EDs of a level 1 and a level 2 trauma center from trauma region Brabant (Amphia Hospital, Breda and Elisabeth-TweeSteden Hospital, location Elisabeth, Tilburg) in a 3-month period (1 November 2013 to 1 February 2014) were included. Patients were contacted and provided with questionnaires 2 months after the ED visit. Recruitment strategy was quasi-randomized as it changed in alternate weeks. Patients attending the ED in the first week were included using the standard recruitment strategy, and patients attending the ED in the second week were approached using the optimized recruitment strategy and so forth.

### Recruitment strategy

In both recruitment strategies, the first set of questionnaires was sent by post by the attended hospital including an information letter, informed consent form, and a return envelope 2 months post-burn. After 2 weeks, non-responders received a reminder letter to participate in the study. Patients were asked to give informed consent for further participation in the study by returning a signed informed consent form and providing personal contact details to the principal researchers. At 6 months, follow-up questionnaires were sent only to patients that gave informed consent. Again, a reminder was sent by post to non-responders after 2 weeks.

Recruitment strategies differed on the following measures: the optimized recruitment strategy used a stamped instead of franked return envelope, added a monetary incentive (€50 coupon raffled among every 50 responders), an additional brochure and sticker of the Dutch Burns Foundation, and a reminder letter including a second copy of the questionnaire (see Table 1). More importantly, in the optimized recruitment strategy, non-responders were contacted by phone by a member of the research team after 2 weeks, as a reminder to participate in the study.

**Table 1:** Comparison of both recruitment strategies

	Standard recruitment strategy	Optimized recruitment strategy
Information letter	✓	✓
Franked return envelope	✓	✗
Stamped return envelope	✗	✓
Information brochure	✗	✓
€50 raffled among each 50 participants	✗	✓
Sticker of DBF on envelope and brochure of DBF	✗	✓
Telephone reminder	✗	✓
Reminder letter	✓	✓
Second copy of questionnaire	✗	✓
Colored ink	✓	✓

*DBF* Dutch Burns Foundation

### Data collection

#### ED hospital database

From the ED hospital databases, data were collected on demographics, burn- and treatment-related characteristics. Socioeconomic status was assessed as an aggregate proxy based on income, education, and work participation in patients’ postal code area, according to the method of the Netherlands Institute for Social Research [17] and classified into quintiles (1 = lowest, 5 = highest). The urgencies on triage are high (Manchester triage system (MTS) [18] red or orange, Emergency severity index (ESI) [19] 1 or 2), middle (MTS yellow, ESI 3), and low (MTS green or blue and ESI 4 or 5). In admitted patients, additional data were derived from the (Dutch) trauma registry.

#### Follow-up by questionnaire

Patients were sent postal questionnaires to collect data on sociodemographic and burn-related characteristics (medical costs, productivity loss (due to work absence), and HRQOL 2 months and 6 months post-burn. Data on pre-burn generic HRQOL was collected 2 months post-burn in adults only; data on burn-specific HRQOL was collected at 6-month follow-up only, to reduce the burden of study data collection for patients.

##### Medical and indirect costs

Data on patients’ extramural medical costs (e.g., physiotherapy) and indirect costs (productivity loss due to work absence) were collected, using the Work and Medical Consumption Questionnaire. This 25-item questionnaire was originally validated to assess productivity loss and medical consumption in patients with psychiatric illness [20]. For this study, the questionnaire was adjusted at some points to make it suitable for burn patients (see Additional file 1).

##### Quality of life

Both generic and burn-specific health-related quality of life were assessed using questionnaires, validated for the Dutch language. Generic HRQOL was assessed using the EuroQol-5D-3L plus cognition dimension (in patients 5 years and older) [21, 22]. In children aged 0–4 years, the Infant and Toddler Quality of Life Questionnaire (ITQOL)-47 was used [23–25].

Burn-specific HRQOL was assessed using the Health Outcomes Burn Questionnaire (HOBQ) for infants and children aged 0–4 years [25], the American Burn Association/Shriners Hospital for Children Burn Outcomes Questionnaire (BOQ) in children aged 5–17 [25], and the Burns Specific Health Scale-brief (BSHS-B) in adults [26, 27].

### Statistical analysis

For both recruitment strategies, response rates were calculated and an odds ratio (OR) with 95% confidence interval (CI) was calculated for the difference in response. Non-response analysis was performed by comparing characteristics of responders versus non-responders, using Fisher’s exact tests (2 × 2 categorical data) or the Fisher’s Fisher-Freeman-Halton exact test (in case of more than two categories), or Mann-Whitney test (non-parametric continuous data) or *t* test (parametric continuous data).

Data was analyzed with SPSS. A *P* value < 0.05 was considered to be statistically significant.

## Results

### Patient and burn characteristics

During the 3-month study period, 87 patients attended one of the two EDs for a burn-related injury. The mean age was 28.4 years and males and females were equally distributed (48.3% vs. 51.7%; see Table 2). One out of four patients lived in an area in the lowest socioeconomic quintile. Scalds caused 34.5% of the burns and flame only accounted for 11.5%. Apart from this, 35.5% of the burn injuries were related to firework. The mean total body surface area (TBSA) burned was 1.6%, and the most frequently affected sites were the hands (40.2%) followed by the head/face (27.6%) (Table 3).

**Table 2:** Characteristics of patients by response^a^ at 2 months

	ED patients	Responders at 2 months	Non-responders at 2 months	*P* value difference by response
	(*n* = 87)	(*n* = 30)	(*n* = 55)	
Mean age (range, SD)	28.4	29.0 (1–64, 18.1)	28.4 (0–83, 20.5)	0.478
Age category (%)^b^				0.975
0–4	10 (12)	3 (10)	6 (11)	
5–17	20 (23)	6 (20)	14 (26)	
18–39	27 (31)	10 (33)	16 (29)	
40–59	25 (29)	10 (33)	15 (27)	
60–79	4 (5)	1 (3)	3 (6)	
80+	1 (1)	0	1 (2)	
Gender: male (%)	45 (52)	18 (60)	26 (47)	0.364
Socioeconomic status, based on postal code				0.677
Lowest quintile 1.	21 (25)	5 (17)	16 (29)	
2.	16 (19)	7 (23)	9 (16)	
3.	19 (22)	7 (23)	12 (22)	
4.	16 (19)	7 (23)	9 (16)	
Highest quintile 5.	13 (15)	4 (13)	9 (16)	
Mean socioeconomic status (SD)^c^	0.013 (1.12)	0.26 (0.97)	0.12 (1.18)	0.155

Percentages rounded up to nearest integer

^a^Response was calculated in 85 patients, excluding 2 patients with undeliverable questionnaires

^b^Age categories 60–79 and 80+ were combined to one category in statistical testing

^c^Mean score socioeconomic status Dutch population = 0.17

*ED* emergency department, *SD* standard deviation

**Table 3:** Characteristics of patients’ burn injuries by response^a^ at 2 months

	ED patients	Responders at 2 months	Non-responders at 2 months	*P* value difference by response
	(*n* = 87)	(*n* = 30)	(*n* = 55)	
Etiology (%)				0.472
Scald	30 (35)	8 (27)	21 (38)	
Flame	10 (12)	3 (10)	7 (13)	
Other	47 (54)	19 (63)	27 (49)	
Firework (%)	30 (35)	11 (37)	16 (29)	0.477
Median TBSA^b^ (25th and 75th percentile)	1.0 (0.2–1.0)	0.5 (0.15–1.0)	1.0 (0.5–2)	0.155
Setting (%)^c^				0.174
Kitchen	14 (16)	2 (7)	12 (22)	
Bathroom	2 (2)	1 (3)	1 (2)	
Garden	1 (1)	0	1 (2)	
Surroundings home	34 (39)	11 (37)	23 (42)	
Work	7 (8)	3 (10)	4 (7)	
Other setting	29 (33)	13 (43)	14 (26)	
Body regions affected (%)^d^				
Head/face	24 (28)	10 (33)	14 (26)	0.460
Neck	5 (6)	1 (3)	4 (7)	0.652
Trunk	15 (17)	6 (20)	9 (16)	0.764
Arm	12 (14)	6 (20)	6 (11)	0.330
Hand	35 (40)	9 (30)	26 (47)	0.167
Leg	9 (10)	6 (20)	3 (6)	0.061
Feet	4 (5)	2 (7)	2 (4)	0.611
Inhalation trauma (suspected) (%)	3 (3)	0	3 (6)	0.549

Percentages rounded up to nearest integer

^a^Response was calculated in 85 patients, excluding 2 patients with undeliverable questionnaires

^b^One missing value (in non-responders)

^c^Bathroom, garden and surroundings home were combined to one category in statistical testing

^d^More than one affected body region per patient is possible

*ED* emergency department, *TBSA* total body surface area

### Response rates

**Fig. 1:**
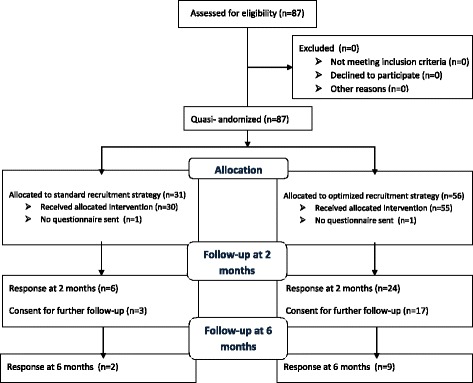
Patient inclusion flow diagram

Totally 85 among 87 patients were sent follow-up questionnaires 2 months after presentation at the ED, using the standard recruitment strategy (*n* = 30) or the optimized recruitment strategy (*n* = 55). Two sets of questionnaires were undeliverable (*n* = 1 standard strategy, no address and *n* = 1 optimized strategy, living abroad). The high number of firework-related burn injuries on New Year’s Eve (*n* = 26) resulted in a higher number of inclusions in the optimized recruitment strategy (see Fig. 1).

There was a higher recruitment and response rate at 2 months in the optimized recruitment strategy (24 out of the 55; 43.6%) than in the standard recruitment strategy (6 out of the 30; 20%) (OR = 3.1, 95% CI = 1.1–8.8) (Additional file 2: Table S1). Out of 30 responders at 2 months, 20 (66.6%) patients gave informed consent for further follow-up at 6 months. At final follow-up at 6 months, 11 (55%) patients returned the questionnaires of whom 9 were initially addressed using the optimized recruitment strategy. The overall response at 6 months was 16.4% (9/55) in the optimized recruitment strategy and 6.7% (2/30) in the standard recruitment strategy (OR = 2.7; 95% CI = 0.6–13.6).

### Non-response analysis

Non-response analyses showed no significant differences in patient or burn injury characteristics between responders versus non-responders at 2 months (see Tables 2 and 3). In addition, no differences in treatment were observed between responders versus non-responders (see Table 4). The majority of patients in both groups received outpatient treatment (76.6% vs. 87.3%), either a single ED contact or multiple outpatient contacts. Additional data on incidence and costs is available upon request.

**Table 4:** Treatment characteristics of burn-related injuries presented at Dutch EDs (*n* = 87), by response^a^

	ED patients *n* = 87		Responders 2 months *n* = 30		Non-responders 2 months *n* = 55		*P* value difference by response
Urgency on triage	*n*	%	*n*	%	*n*	%	0.878
High	18	20.7	7	23.3	10	18.2	
Middle	30	34.5	10	33.3	19	34.5	
Low	39	44.8	13	43.3	26	47.3	
Specialized burn care^b^	12	13.8	4	13.3	7	13.0	1.00
Treatment trajectory							
Single ED contact	36	41.4	10	33.3	25	45.5	0.346
Multiple outpatient contacts	36	41.4	13	43.3	23	41.8	
Admission	15	17.2	7	23.3	7	12.7	
Related ED follow-up visits							
1	72	82.8	23	76.7	47	85.5	0.376
2 or more	15	17.2	7	23.3	8	14.5	
Related hospital follow-up visits							
0	38	43.7	12	40.0	24	43.6	0.820
1 or more	49	56.3	18	60.0	31	56.4	

^a^Response was calculated in 85 patients, excluding 2 patients with undeliverable questionnaires

^b^Included patients admitted to specialized burn care, as well as patients treated as an outpatient in specialized burn care. One missing value (in non-responders (*n* = 1))

*ED* emergency department

## Discussion

This study aimed to improve and evaluate patient recruitment strategy and follow-up methods in an ED burn population. Using strategies to optimize recruitment yielded higher recruitment and response rate at 2 months (43.6 vs. 20.0%, OR = 3.1, 95% CI = 1.1–8.8). This might seem as a self-evident result; however, the extra labor and costs that the optimized recruitment strategy entailed must be taken into account, when assessing the feasibility of a larger scale study including multiple EDs. Moreover, in the optimized strategy, local researchers are required in all participating hospitals for the reminder by telephone in non-responders; as for legal reasons, it is not allowed to transfer patients’ contact information to coordinating researchers.

A crucial outcome of our study was the low participation and response rate, also in the optimized recruitment strategy. This could be explained partly by the postal recruitment strategy, a timing of the assessment at 2 months post-burn in combination with relatively small burns. Due to the low incidence of burn injuries, in combination with a large number of staff and the short time window within which patients at the ED can be recruited, onsite recruitment was deemed not feasible.

Next, the relatively low socioeconomic status of burn injury patients can add to the low response rates in burn injury populations. Hutchings [28] reported significant higher levels of non-response in patients from the most deprived quintiles of socioeconomic status.

Recruitment and follow-up at specialized burn centers is less problematic, also in outpatient clinics, as incidence of burn injuries is high, burn injury is frequently more severe, and often a dedicated treatment team with the support of a research team is available, resulting in direct onsite informed consent conversation, instead of delayed postal recruitment. The optimized follow-up strategy ideally should always be applied to minimize attrition.

Previous studies in ED patients after general injuries showed similar responses with a 37% to 43% response rate on a first postal questionnaire on costs and generic quality of life assessments post-injury. In these studies, patients with a hospital admission were oversampled [9, 10]. Finlay et al. [29] reported a response rate of 63% at least 6 months post-burn, after intensive follow-up including multiple phone calls, in patients with minor burns treated in a burn center [29]. The authors needed 180 h, i.e., more than 2 h per patient per follow-up to achieve this response rate. This response rate is probably not realistic in an ED population, with small-sized burns (mean TBSA < 2%). Gabbe et al. [30] reported a dramatic decrease over time in response rates in patients admitted to a burn center who were followed up, which decreased from 64% at 1-month follow-up to 21% at 24-month follow-up [30]. Recently, Varner showed a lower attrition rate in ED patients using text messaging reminders. These text messaging reminders were sent only in case of unsuccessful telephone contact. Alternatively, emails can be sent [31].

Review papers on recruitment and retention in emergency medicine studies [32] as well as in clinical trials in general [33] underscore the need for optimal strategies but also the lack of evidence what works in recruitment and follow-up. Thus, recruitment and response rates are a major issue in burn research and in other fields and deserve continued attention to optimize questionnaire research.

A strength of this study was the completeness of data on epidemiology of ED burns. Data on characteristics of injury and treatment could be adequately retrieved from ED electronic medical records and the Dutch trauma registration (in admitted patients). Next to this, information on specialized burn care was available to participating researchers from the burn center itself.

A limitation of our study was the limited number and unequal distribution of patients across the recruitment strategies. The recruitment strategy that changed across alternate weeks resulted in an unbalanced patient distribution across strategies because of a peak on New Year’s Eve (*n* = 26). Beforehand, alternative recruitment schemes were discussed but not considered feasible with regard to the future larger scale study. However, future studies should use another design; perhaps recruitment can alternate after every ten patients, to prevent unbalanced groups. Also, sample size calculation should be included to optimize study efficiency. As our study was designed as a feasibility study, no sample size calculation was performed.

Next, response rates remained limited, even in the optimized recruitment strategy (43.6%). This could introduce selection bias. Earlier ED studies reported similar response rates of 37–43% but sampled the more severe patients (i.e., used a stratified patient selection based on injury category and overrepresentation of admitted patients and patients with an expected continued treatment) [9, 10]. This stratification method is supported by Finlay et al., who found that loss to follow-up in patients with minor burns and burns affecting the upper limb was an indicator of good recovery and concluded that follow-up of these patients is unnecessary [29]. Recently, this group developed a prognostic model for tailoring burn care to more severe patients. Male gender, conservative management, upper limb burn, and good burn-specific HRQOL within 1 month of burn were significant predictors of good outcome at 6 months and beyond. However, these proposals need further study [34]. We could not address this issue in our analysis because of limited follow-up data.

We did not perform direct recruitment at the ED but contacted patients 2 months post-burn. Direct recruitment was judged not feasible because the low frequency of burn injuries and the large number of staff. Probably, a shorter time from injury to first study contact would have increased response. In future studies, the possibilities of a timelier, local recruitment need to be explored. In future studies, an optimized recruitment strategy, shortly after injury, in combination with the selection of more severe burn patients for follow-up can contribute to a further increase in response rates to an acceptable level. We propose the use of an algorithm based on burn severity (e.g., TBSA) and treatment trajectory for the selection of the majority of follow-up patients. The length of the questionnaires must also be minimized, to reduce the burden for patients and lower the barrier for participation in follow-up. Also, web-based data collection must be considered to optimally facilitate potential participants. Furthermore, the study period should last at least a full calendar year to cover all seasonal fluctuations in burn injuries presenting at EDs.

## Conclusion

This study demonstrated that response rates can be increased with an optimized, but more labor-intensive, and thus more expensive recruitment strategy, although further optimization of recruitment and follow-up is needed. When abovementioned points of improvement are implemented in a larger scale study, we assume it is feasible to assess the real burden of disease in this population including HRQOL and extramural costs.

## Additional files

Additional file 1: Work and Medical Consumption questionnaire. (DOCX 44 kb)

Additional file 2: Table S1. Detailed response analysis by recruitment strategy (DOCX 16 kb)

## Abbreviations

BOQ: Burn Outcomes Questionnaire
BSHS-B: Burns Specific Health Scale-brief
CI: Confidence interval
ED: Emergency department
EQ-5D: EuroQol-5D-3L
ESI: Emergency severity index
HOBQ: Health Outcomes Burn Questionnaire
HRQOL: Health-related quality of life
ITQOL: Infant and Toddler Quality of Life Questionnaire
LIS: Dutch Injury Surveillance System
MTS: Manchester triage system
OR: Odds ratio
SD: Standard deviation
TBSA: Total body surface area
