# Supplementary material for: Follow-up in patients with a burn-related emergency department visit: a feasibility study
Source: Burns Trauma. 2017 Nov 8;5:35. doi: 10.1186/s41038-017-0100-1 (PMC5678580; doi:10.1186/s41038-017-0100-1)
Supplement: Supplementary file 1 — Work and Medical Consumption questionnaire. (DOCX 44 kb) [file 41038_2017_100_MOESM1_ESM.docx]

**Work and Medical Consumption questionnaire**

**Questionnaire about your contacts with health care and work during the past 3 months**

**Explanation**

**Please read this first!**

**Who is this questionnaire for?**

This questionnaire is for you. There are some options:

- You have received this form from your (family) doctor or from the hospital.
- You have received this form by post with your name posted on the envelope.

**You cannot fill in the form on your own?**

If you are unable to fill in the form on your own, maybe someone can help you, for example a member of your family.

**What is the questionnaire about?**

The questionnaire is about contacts with health care providers and about work during the last three months. We will start with a number of general questions, for example regarding your date of birth and gender. Next we ask about your contacts with healthcare providers. And about work and what you do at home.

**How long does it take to fill in the form?**

It takes about 30 minutes to fill in the form.

**How should you fill in the form?**

- Start with the first question and follow the numbering.
- Put 1 **x** in the question box, unless the question states that you may put more than 1 **x**.
- For some of the questions you may fill in a number or otherwise on the dotted line.
- There are no wrong answers.

**Do you want to change an answer?**

- Cross out the old answer.
- Put an **x** in the new answer box.
- Put an arrow in front of the new answer.

🗙 old answer

🡪 🗙 new answer

**What happens to your answers?**

Your answers will be used for research. Only the researchers will see your answers. That means therefore no one else.

The researchers do not write your name anywhere. And they will not tell anyone that you have participated in this research project.

**We greatly appreciate that you are willing to fill in this form for us!**

***What are the consequences of your burn injury on work?***

Are you younger the 70 years of age? Answer the following questions. If not, continue with question 8.

**1. What is the highest degree in education that you have achieved?** Look for your highest degree in education and fill in an **x** in the box.

- I never finished school or training programma
- Primary school or elementary school
- Junior vocational education
- Lower general secondary school
- Intermediate vocational education
- Higher general secondary education
- School for higher vocational education
- University
- I achieved another degree, namely .........................................................................

**2. Did you had a paying job before the burn injury?**

You were employed or self-employed.

- Yes
- No

Did you answer ‘Yes’? Continue with question 3 to 7. If not, continue with question 8.

**3. How many days a week did you work prior to your burn injury?**

|  |  |  |  | Days a week |
| --- | --- | --- | --- | --- |
|  |  |  |  |  |
|  |  |  |  |  |

**4. How many hours a week did you work prior to your burn injury?** Count only the hours that you get paid.

|  |  |  |  | Hours a week |
| --- | --- | --- | --- | --- |
|  |  |  |  |  |
|  |  |  |  |  |

**5. Have you missed work in the last 3 months as a result of your burn injury?**

- Yes
- No

Did you answer ‘Yes’? Continue with question 6 and 7. If not, continue with question 8.

**6. Do you miss work at this moment?**

- Yes
- No

**7. How many work days did you miss work in total as a result of your burn injury?**

|  |  |  |  | Work days |
| --- | --- | --- | --- | --- |
|  |  |  |  |  |
|  |  |  |  |  |

***What are the consequences of your burn injury on work of your partner?***

Is your partner younger the 70 years of age? Then answer the following questions. If not, continue with question 15.

**8. What is the highest degree in education that your partner achieved?** Look for your partner highest degree in education and fill in a **x** in the box.

- He/she never finished school or training programma
- Primary school or elementary school
- Junior vocational education
- Lower general secondary school
- Intermediate vocational education
- Higher general secondary education
- School for higher vocational education
- University
- He/she achieved another degree, namely............................................................

**9. Did your partner had a paid job prior to your burn injury?**

He/she was employed or self-employed.

- Yes
- No

Did you answer ‘Yes’? Continue with question 10 to 14. If not, continue with question 15.

**10. How many days a week did your partner work prior to your burn injury?**

|  |  |  |  | Days a week |
| --- | --- | --- | --- | --- |
|  |  |  |  |  |
|  |  |  |  |  |

**11. How many hours a week did your partner work prior to your burn injury?** Count only the hours that your partner get paid.

|  |  |  |  | Hours a week |
| --- | --- | --- | --- | --- |
|  |  |  |  |  |
|  |  |  |  |  |

**12. Has your partner missed work in the last 3 months as a result of your burn injury?**

- Yes
- No

Did you answer ‘Yes’? Continue with question 13 to 14. If not, continue with question 15.

.

**13. Does your partner miss work at this moment as a result of your burn injury?**

- Yes
- No

**14. How many work days did your partner miss work in total as a result of your burn injury?**

|  |  |  |  | Work days |
| --- | --- | --- | --- | --- |
|  |  |  |  |  |
|  |  |  |  |  |

***Questions about your travel to the burn centre***

**15. What is the distance (single way) to the burn centre?**

|  |  |  |  | Kilometer |
| --- | --- | --- | --- | --- |
|  |  |  |  |  |
|  |  |  |  |  |

**How do you travel?**

- Car
- Public transport
- Other, namely

**16. What is the average travel time per single way to the burn centre?**

|  |  |  |  | Minutes |
| --- | --- | --- | --- | --- |
|  |  |  |  |  |
|  |  |  |  |  |

## Questions about medical consumption outside the burn centre

**Explanation**

We would like to know which doctors you have consulted during the last three months outside of the burn centre. This is about consultations related to your burn injury. Also other health care providers have to be counted. For example a physiotherapist.

What consultations must be counted?

- Control visits
- Appointments because you had a physical or mental problem
- Visits of the doctor at your house
- Consultations by telephone with your doctor
- Telephone calls for a prescription

What should not be counted?

- Appointments for someone else, for example for your child
- Telephone calls for making an appointment

Are you unsure about the exact number of consultations? Please fill in how many consultations you have had approximately.

**17. Were you admitted to a residential care or nursing home after discharge from the burn centre?**

|  | □ | Yes, |  |  | days |  |
| --- | --- | --- | --- | --- | --- | --- |
|  |  |  |  |  |  |  |
|  |  |  |  |  |  |  |
|  | □ | No | | | | |
|  |  |  |  |  |  |  |

**18. Were you admitted to a rehabilitation centre home after discharge from the burn centre?**

|  | □ | Yes, |  |  | days |  |
| --- | --- | --- | --- | --- | --- | --- |
|  |  |  |  |  |  |  |
|  |  |  |  |  |  |  |
|  | □ | No | | | | |
|  |  |  |  |  |  |  |

**19. Did you visit your general practioner for your burn injury during the last three months?**

|  | □ | Yes, |  |  | times |  |
| --- | --- | --- | --- | --- | --- | --- |
|  |  |  |  |  |  |  |
|  |  |  |  |  |  |  |
|  | □ | No | | | | |
|  |  |  |  |  |  |  |

**20. Did you visit a physiotherapist for treatment related to your burn injury (or Caesar therapist, Mensendieck therapist or a manual therapist)?** Add up all appointments with these therapists.

Physiotherapy inside the burn centre, nursing home or rehabilitation centre do not count

|  | □ | Yes, |  |  | times |  |
| --- | --- | --- | --- | --- | --- | --- |
|  |  |  |  |  |  |  |
|  |  |  |  |  |  |  |
|  | □ | No | | | | |
|  |  |  |  |  |  |  |

**21. Did you receive home care for your burn injury during the last three months?**

For example: nursing, personal care or housekeeping and home help.

- Yes
- No

Did you answer ‘Yes’? Continue with question the next questions. If not, continue with question 24.

**22. How many weeks did you receive this home care during the last three months?**

|  |  |  |  | Weeks |
| --- | --- | --- | --- | --- |
|  |  |  |  |  |
|  |  |  |  |  |

**23. For how many hours on average did you receive home care during these weeks?**

|  |  |  |  | Hours a week |
| --- | --- | --- | --- | --- |
|  |  |  |  |  |
|  |  |  |  |  |

**24. Did you, as a result of your burn injury, visited any other health care provider during the last three months?**

For example: psychologist, speech therapist, social worker, etc. healthcare providers inside the burn centre, nursing home or rehabilitation centre do not count.

- Yes
- No

Did you answer ‘Yes’? Please answer the last question.

**25. Which other healthcare providers did you visit?**

|  |  | Discription healthcare provider | Times, number of weeks etc. |
| --- | --- | --- | --- |
|  |  |  |  |
|  | □ |  |  |
|  | □ |  |  |
|  | □ |  |  |
|  | □ |  |  |

**Thank you very much!**
