# Supplementary material for: Follow-up in patients with a burn-related emergency department visit: a feasibility study
Source: Burns Trauma. 2017 Nov 8;5:35. doi: 10.1186/s41038-017-0100-1 (PMC5678580; doi:10.1186/s41038-017-0100-1)
Supplement: Supplementary file 2 — Detailed response analysis by recruitment strategy (DOCX 16 kb) [file 41038_2017_100_MOESM2_ESM.docx]

**Additional file 2: Table S1. Detailed response analysis by recruitment strategy**

|  | Total cohort | Standard recruitment strategy | Optimized recruitment strategy | p-value |
| --- | --- | --- | --- | --- |
| Eligible patients | 87 | 31 | 56 |  |
| Questionnaires sent | 85 | 30 | 55 |  |
| Not deliverable | 2 | 1 | 1 |  |
|  |  |  |  |  |
| Returned at 2 months | 30 | 6 | 24 |  |
| Response rate 2 months | 35.0% | 20.0% | 43.6% | 0.052 |
|  |  |  |  |  |
| Questionnaires sent | 20 | 4 | 16 |  |
| Not deliverable (no informed consent or no contact information given) | 10 | 2 | 8 |  |
| Returned at 6 months | 11 | 2 | 9 |  |
| Response rate 6 months | 55.0% | 50.0% | 56.3% |  |
| Overall response rate 6 months | 12.9% | 6.7% | 16.4% |  |
